# Transcriptomics Analysis of Apple Leaves in Response to *Alternaria alternata* Apple Pathotype Infection

**DOI:** 10.3389/fpls.2017.00022

**Published:** 2017-01-20

**Authors:** Longming Zhu, Weichen Ni, Shuai Liu, Binhua Cai, Han Xing, Sanhong Wang

**Affiliations:** ^1^Department of Horticulture, Nanjing Agricultural UniversityNanjing, China; ^2^National Key Laboratory of Crop Genetics and Germplasm Enhancement, Department of Agricultural, Nanjing Agricultural UniversityNanjing, China

**Keywords:** transcriptomic, apple (*Malus* × *domestica* Borkh.cv. “Starking Delicious”), *Alternaria alternata* apple pathotype, infection, defense response

## Abstract

Alternaria blotch disease of apple (*Malus* × *domestica* Borkh.), caused by the apple pathotype of *Alternaria alternata*, is one of the most serious fungal diseases to affect apples. To develop an understanding of how apples respond to *A. alternata* apple pathotype (AAAP) infection, we examined the host transcript accumulation over the period between 0 and 72 h post AAAP inoculation. Large-scale gene expression analysis was conducted of the compatible interaction between “Starking Delicious” apple cultivar and AAAP using RNA-Seq and digital gene expression (DGE) profiling methods. Our results show that a total of 9080 differentially expressed genes (DEGs) were detected (>two-fold and FDR < 0.001) by RNA-Seq. During the early phase of infection, 12 h post inoculation (HPI), AAAP exhibited limited fungal development and little change in the transcript accumulation status (950 DEGs). During the intermediate phase of infection, the period between 18 and 36 HPI, increased fungal development, active infection, and increased transcript accumulation were detected (4111 and 3838 DEGs detected at each time point, respectively). The majority of DEGs were detected by 72 HPI, suggesting that this is an important time point in the response of apples' AAAP infection. Subsequent gene ontology (GO) and pathway enrichment analyses showed that DEGs are predominately involved in biological processes and metabolic pathways; results showed that almost gene associated with photosynthesis, oxidation-reduction were down-regulated, while transcription factors (i.e., WRKY, MYB, NAC, and Hsf) and DEGs involved in cell wall modification, defense signaling, the synthesis of defense-related metabolites, including pathogenesis-related (PRs) genes and phenylpropanoid/cyanoamino acid /flavonoid biosynthesis, were activated during this process. Our study also suggested that the cell wall defensive vulnerability and the down-regulation of most PRs and HSP70s in “Starking Delicious” following AAAP infection might interpret its susceptible to AAAP.

## Introduction

Alternaria blotch disease of apple, caused by the *Alternaria alternata* apple pathotype (AAAP), is one of the most serious fungal diseases affecting apples globally, especially in East Asia (Saito et al., 2001). This disease affects apple tree growth and production via the infection of leaves, young shoots, and fruits and leads to marked declines in tree vigor. Initial lesions appear first on apple leaves in late spring or early summer as small, round, black-colored spots that gradually enlarging to 2–5 mm in diameter and have a brownish purple border. These lesions may coalesce or undergo secondary enlargement and become irregular and much darker, acquiring a “frog-eye” appearance (Li et al., 2013). Severe AAAP infection can result in 60–80% defoliation (Filajdic and Sutton, 1991), leading to premature fruit drop. However, both the rate of occurrence and severity of *Alternaria* leaf blotch vary between apple cultivars. For example, “Jonathan,” “Jonagold,” and “Gala” are resistant to AAAP, while the “Starking Delicious” and “Indo” varieties are highly susceptible to infection (Abe et al., 2010). Thus, extensive breeding research has been carried out to understand genetics of the disease resistance as well as the underlying mechanism in order that new cultivars resistant to the pathogen can be produced. Little progress has been made in these areas, however, mainly because of our limited genetic understanding of response to the pathogen and the long apple breeding cycle. Application of fungicides is currently the main method to control AAAP; however, continuous and repetitive use of these chemicals can result in the individual selection of resistant pathogen and potentially pollutes the environment. Thus, an improved understanding of the defense mechanisms used by apples in response to AAAP will contribute to the design of new and safer control strategies, as well as aiding in the development of resistant cultivars.

Plants have evolved a number of strategies to effectively combat invasion of pathogens, involving a large number of physiological responses including hypersensitive reaction (HR), cell walls modifications, and the production of antimicrobial proteins and metabolites (Cohn et al., 2001). These physiological responses are associated with the reprogramming of numerous defense-related genes and transcription factors. Thus, as technology has advanced, approaches in comparative “omics” have greatly contributed to the effort of defining gene and protein functions and our understanding of their expression and changes in accumulation during plant-pathogen interactions (Mathioni et al., 2011; Zhao et al., 2014). Enormous datasets have been derived from model plant organisms, enabling the identification of defense-related genes and proteins (Thilmony et al., 2006; Mukherjee et al., 2010; Proietti et al., 2013). Apple is not just an agronomically important crop; it is also a model species within Rosaceace (Shulaev et al., 2008). Although, several studies to date have analyzed apple-pathogen interactions (Sarowar et al., 2011; Vilanova et al., 2014), this study is the first global transcription analysis of apple in response to AAAP.

In this study, we conducted a large-scale gene expression analysis of the “Starking Delicious” apple cultivar in response to AAAP infection using RNA-Seq and digital gene expression (DGE) methods. Use of these approaches enabled us to gain molecular insights into the changes in transcriptional expression that are associated with apple defense to the fungus, as well as the transcriptional response of “Starking Delicious” to AAAP infection, and the possible reasons that underlie its susceptibility to this pathogen.

## Materials and methods

### Plant materials, AAAP culture, and inoculation method

Two-year-old “Starking Delicious” cultivar apple plants grafted on *Malus robusta* stocks were grown in a greenhouse at Nanjing Agricultural University, located in Nanjing, Jiangsu Province, China. The AAAP fungus was expanded on a potato dextrose agar (PDA; 200 g potato extract, 20 g dextrose, 20 g agar, 1 L water) medium for 5 days at 26°C under dark conditions. The inoculation method was carried out according to the protocol described previously (Abe et al., 2010); the fourth and fifth youngest opened leaves from shoot tips were collected from plants and inoculated with mycelia together with PDA medium punched using a hole punch (diameter = 5 mm). In each treatment, leaves were inoculated with six cakes of mycelium applied to both side of the midrib of the abaxial leaf surfaces; the mock-inoculation of leaves using PDA medium cakes instead of mycelia was also carried out as a control. Thus, five groups of leaves were inoculated with mycelia or PDA cakes at 36, 18, 6, and 4 h intervals and then incubated at 25°C under a 14 h light/10 h dark cycle in sterilized plastic chambers. The groups were then sampled simultaneously when the first set of inoculated leaves reached the 72 h post inoculation (HPI) time point; these five groups represent the five infection stage, 72, 36, 18, 12, and 8 HPI. Leaves sampled at 8 and 12 HPI were used to visualize new hypha development and necrotic tissue. Trypan blue stain is as previously described (Koch and Slusarenko, 1990). All microscopic observations were conducted using a compound microscope.

Leaf samples were placed rapidly into liquid nitrogen and then stored at −70°C for RNA extraction. Each stage contained three parallel leaves from three apple trees that represented three biological replicates.

### Total RNA extraction, library construction, and sequencing

Total RNA was isolated using a cetyltrimethyl ammonium bromide method (Chang et al., 1993; Pavy et al., 2008). Equal quantities of RNA from three biological replications at each stage were pooled to construct a complementary DNA (cDNA) library. Oligo-(dT) magnetic beads were used to isolate poly-(A) messenger RNA (mRNA) from total RNA, and mRNA was fragmented in fragmentation buffer. Using these short fragments (≈200 bp) as templates, random hexamer-primers were then used to synthesize first-strand cDNA, while second-strand cDNA was synthesized using buffer, dNTPs, RNaseH, and DNA polymerase I. Short double-stranded cDNA fragments were purified using a QiaQuick PCR extraction kit (Qiagen, Venlo, Netherlands), resolved with elution buffer for end reparation and the addition of poly (A), before being ligated to sequencing adapters. Subsequent to purification using agarose gel electrophoresis, suitable fragments were enriched via PCR amplification, and libraries were sequenced on the Illumina HiSeq™2000 platform at the Beijing Genomics Institute (Shenzhen, China; http://www.genomics.cn/index), following the manufacturer's protocols.

### RNA-Seq data analysis

Raw reads from the image data output from the sequencing machine were generated by Base Calling and saved in FASTQ format. Clean reads were generated by removing reads with adaptors, reads where the number of unknown bases was more than 10%, and low-quality reads (the percentage of the low-quality bases with which value ≤5 was more than 50% in one read).

Clean reads were then aligned to reference sequences using the software SOAPaligner/soap2 (Li et al., 2009) with no more than two mismatches were allowed in each alignment. The *Malus* × *domestica* Borkh. cv. “Golden Delicious” genome assemblies as well as related gene annotations were downloaded from the Phytozome V9.0 database (ftp://ftp.jgi-psf.org/pub/compgen/phytozome/v9.0/Mdomestica/assembly), (ftp://ftp.jgi-psf.org/pub/compgen/phytozome/v9.0/Mdomestica/annotation).

Gene expression levels were calculated using the “reads per kb per million reads” (RPKM) method (Mortazavi et al., 2008), while differential expressed genes (DEGs) were analyzed as described previously (Audic and Claverie, 1997). In addition, the false discovery rate (FDR) ≤ 0.001 and an absolute value of $\log_{2}^{\left|\text{Ratio}\right|}$ ≥ 1 were used as the thresholds to judge the significance of differences in gene expression.

Gene ontology (GO) categories were assigned to all genes via a BLASTX hit using the Blast2GO software. DEGs were first mapped to GO terms using a standard database (http://www.geneontology.org/), gene numbers for each term were calculated, and GO terms significantly enriched in DEGs compared to the background genome were determine with a hypergeometric test. All calculated *P*-values were then subjected to Bonferroni Correction, using a corrected *P* ≤ 0.05 as the threshold. GO terms that fulfilled this criterion were defined as significantly enriched in DEGs. On the basis of the Kyoto Encyclopedia of Gene and Genome (KEGG), pathway enrichment was then analyzed using the same method, while the WEGO (http://wego.genomics.org.cn/cgi-bin/wego/index.pl) software was used for functional classification of GO terms following DEG GO annotation.

### qRT-PCR verification

Gene-specific primers for qRT-PCR were designed using the Beacon Designer 7.0 program (Premier Biosoft International, California, USA) and are listed in Table S1. Template cDNAs were synthesized using M-MLV reverse transcriptase (Promega, USA) from 1.0 μg of total RNAs following the manufacturer's instructions. 2 × SYBR-Green I RT-PCR Master Mix (Takara, Japan) was used as the labeling agent, while *tubulin* from *M. domestica* served as the internal reference gene. These reactions were performed on an Applied biosystems 7300 Real Time PCR System, with the reaction mixture (20 μL) containing 10 μL 2 × Master Mix, 10 μmol·L^−1^ forward and reverse primers (0.4 μL each), and 1 μL template cDNA. The PCR program in this case was 95°C for 1 min, followed by 40 cycles at 95°C for 20 s, 60°C for 20 s and 72°C for 40 s. Three independent biological replicates were performed for each sample, while the relative expression levels of the selected unigenes were calculated using the relative 2^−ΔCT^ method (Livak and Schmittgen, 2001). The results represented in this case represent mean standard deviations for the three experimental replicates.

## Results and discussion

### Changes in symptoms following AAAP infection

**Figure 1:**
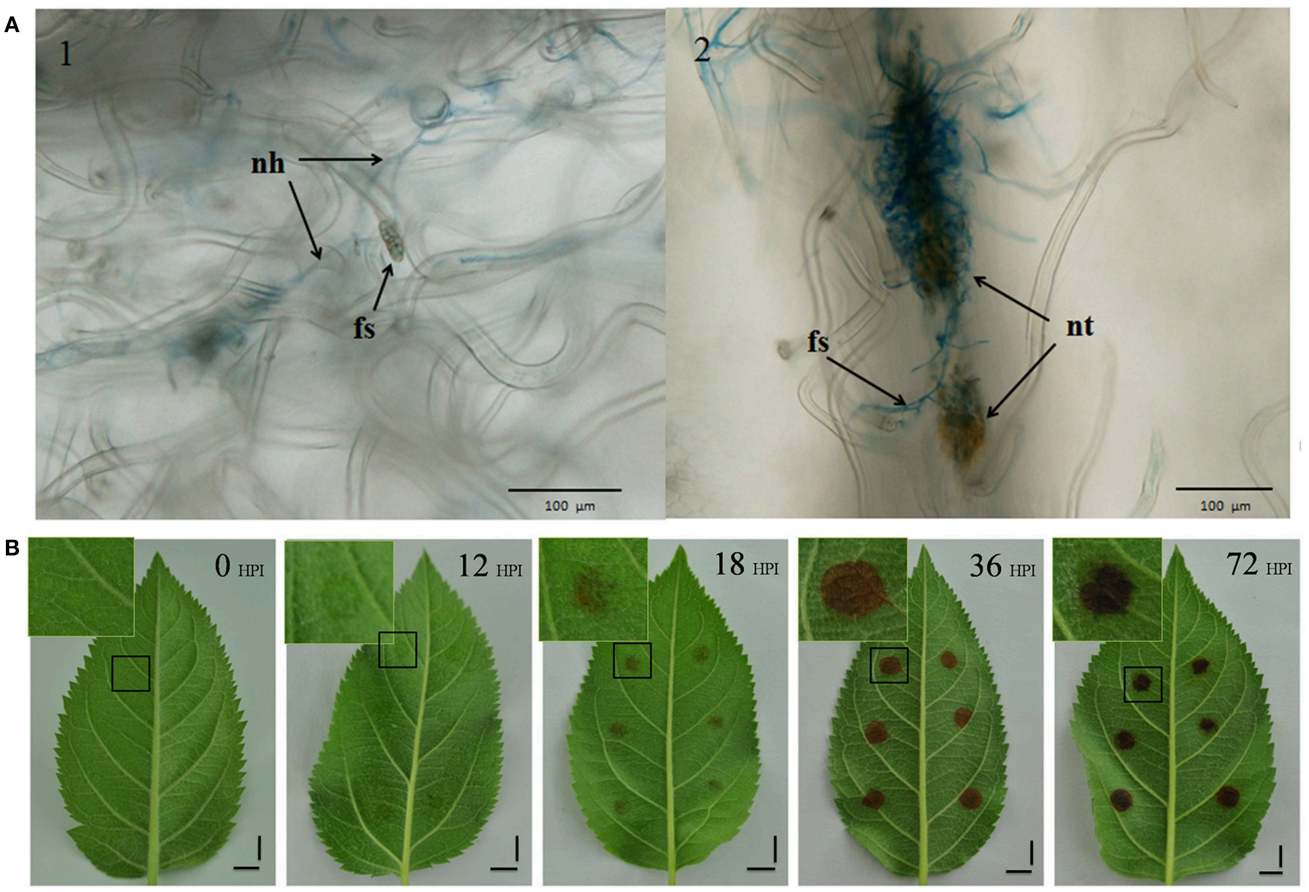
**Symptoms of AAAP infection. (A)** Infected leaves selected from 8 HPI (Panel 1) and 12 HPI (Panel 2) stained with trypan blue and imaged with optical microscopy. nh, new hyphae; fs, fungal spores; nt, necrotic tissue. Scale bar = 100 μm. **(B)** Changes in symptoms in “Starking Delicious” apple leaves after infection with *A. alternata* AP. Images show the alternaria blotch at 0, 12, 18, 36, and 72 HPI, with enlarged views of portions of damaged tissues in insets. Scale bar = 1 cm.

We conducted an initial microscope study to determine an appropriate initial sampling time, staining inoculated leaves with trypan blue. The observations made during this study show that while some spores developed new hyphae by 8 HPI, no necrotic tissue formed by this time point. However, because necrotic tissue was present by 12 HPI, obviously infected by new hyphae (Figure 1A), this point was used as our first sampling time.

We recorded a series of macroscopic observations marking the progress of disease on the inoculated leaves (Figure 1B); leaf areas containing mycelial “cakes” appeared to be water soaked by 12 HPI even though the disease symptoms were not obvious, while blotches comprising damaged tissues appeared to be weakly brown by 18 HPI. Observation confirm that blotch size increased and deepened in color over the subsequent 18 h; following this, there was no further increase in blotch size but color deepened from brown to black.

### RNA-Seq data and apple DGE profiles in response to AAAP infection

**Figure 2:**
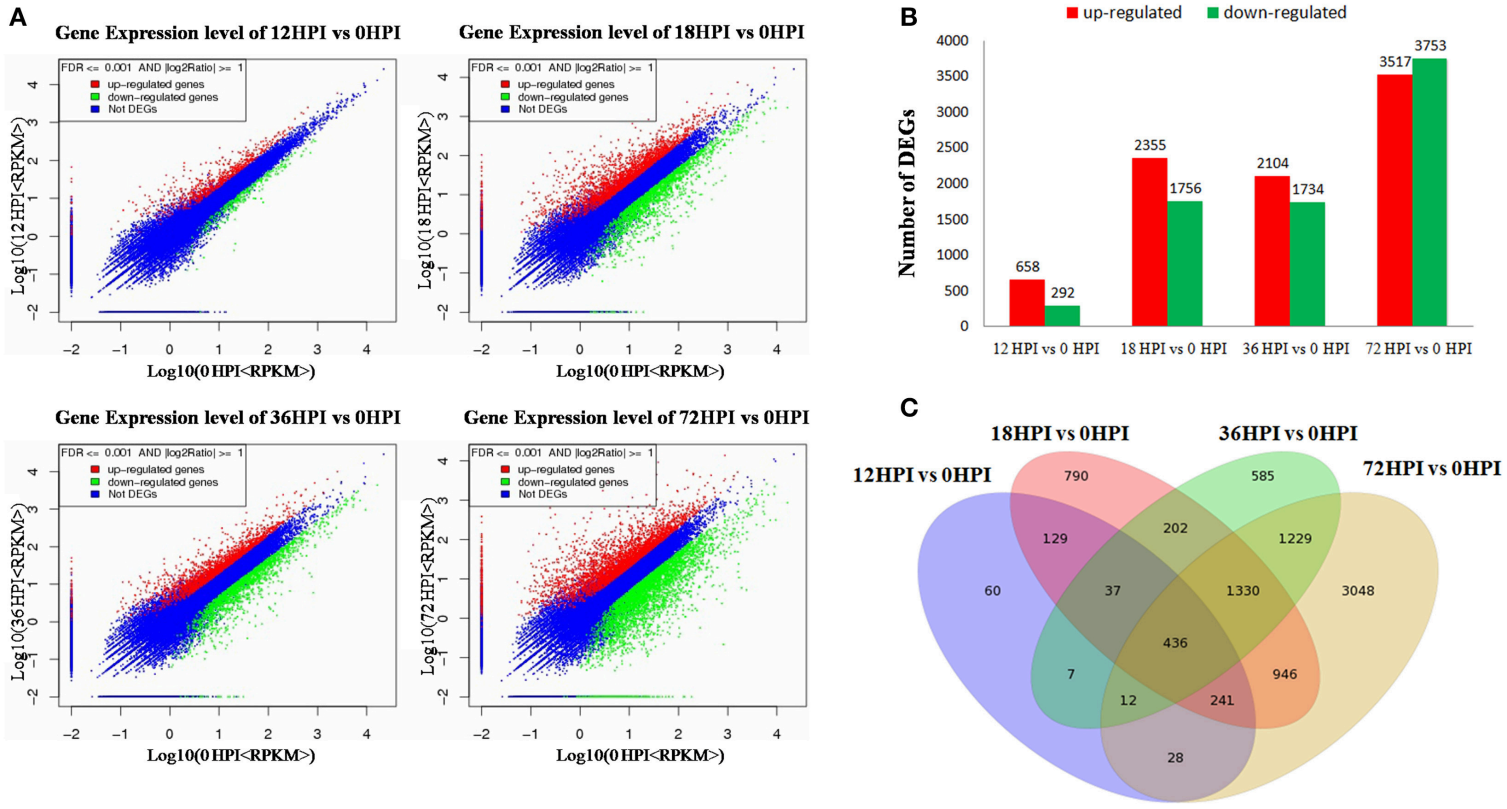
**DEGs between samples. (A)** Scattered plot of differential expression. **(B)** Numbers of DEGs compared between two samples (i.e., 12 vs. 0 HPI, 18 vs. 0 HPI, 36 vs. 0 HPI, 72 vs. 0 HPI, and with 0 HPI as the control). DEGs are shown in red (up-regulated) and green (down-regulated). **(C)** Venn diagram analysis of the DEGs in “Starking Delicious” apple leaves after inoculation with *A. alternata* AP.

According to the macroscopic and microscopic observation, we constructed four RNA libraries, i.e., 12, 18, 36, and 72 HPI, representative of four treatments of leaves inoculated by AAAP at 12, 18, 36, and 72 h. In addition, we mixed the mock-inoculated leaves of four time points into one sample as control (0 HPI). Five RNA libraries were analyzed using RNA-Seq method in combination with comparative DGE profiling. The sequencing raw data set has been deposited in National Center for Biotechnology Information Sequence Read Archive database (accession number accession number SRP091754). Approximately 11.96~12.61 million of raw RNA-Seq reads were produced for each sample. After filtering the dirty reads, 10.03~10.70 million and 7.83~8.36 million were mapped, of which 6.40~6.90 million and 5.38~5.61 million reads were uniquely mapped to reference genomes (Table S2-1) and specific genes (Table S2-2), respectively. On the basis of these mapped reads, a total of 41,022 gene expression levels were calculated using the RPKM method (Figure 2A, Excel S1).

Gene expression comparisons were performed between the AAAP-infected and control samples; compared with 12 and 0 HPI libraries (12 vs. 0 HPI), 950 DEGs were identified, of which 658 were up-regulated and 292 were down-regulated. In contrast, when the 18 and 0 HPI libraries were compared (18 vs. 0 HPI), 4111 DEGs were generated with 2355 up-regulated and 1756 down-regulated, respectively, while 3838 DEGs were identified when the 36 and 0 HPI libraries were compared (36 vs. 0 HPI), of which 2104 were up-regulated and 1734 were down-regulated, respectively. Out of all comparisons, however, the largest number of DEGs, 7270, were found when the 72 with 0 HPI libraries were compared (72 vs. 0 HPI) of which 3517 were up-regulated and 3753 were down-regulated, respectively (Figure 2B, Excel S2). Results showed that these transcriptomic changes do exhibit some shared characteristics when compared with fungal development and symptom phenotypes, including limited fungal development and little change in the transcript accumulation by 12 HPI. At intermediate stage, 18 and 36 HPI, increases in the level of fungal development, active infection, and increased transcript accumulation are seen, while the majority of DEGs were detected by 72 HPI which suggests that this time, in particular, is important for the apple in response to AAAP infection.

When these comparative results are illustrated as Venn diagram, it is clear that both unique and shared DEGs occur between, and among, pairs (Figure 2C). For example, 890 DEGs (93.68% of the total) in 12 HPI vs. 0 HPI, 3321 DEGs (81.79% of the total) in 18 vs. 0 HPI, 3253 DEGs (84.76% of the total) in 36 vs. 0 HPI and 4222 DEGs (59.07% of the total) in 72 vs. 0 HPI shared with the other compared libraries respectively, moreover, 436 DEGs are shared across all comparisons. These results suggest that as the pathogen infection progresses, more genes become involved in defense response, and a large number of genes that respond early to pathogen infection also function subsequently.

### Functional annotation of differentially expressed genes (DEGs)

**Figure 3:**
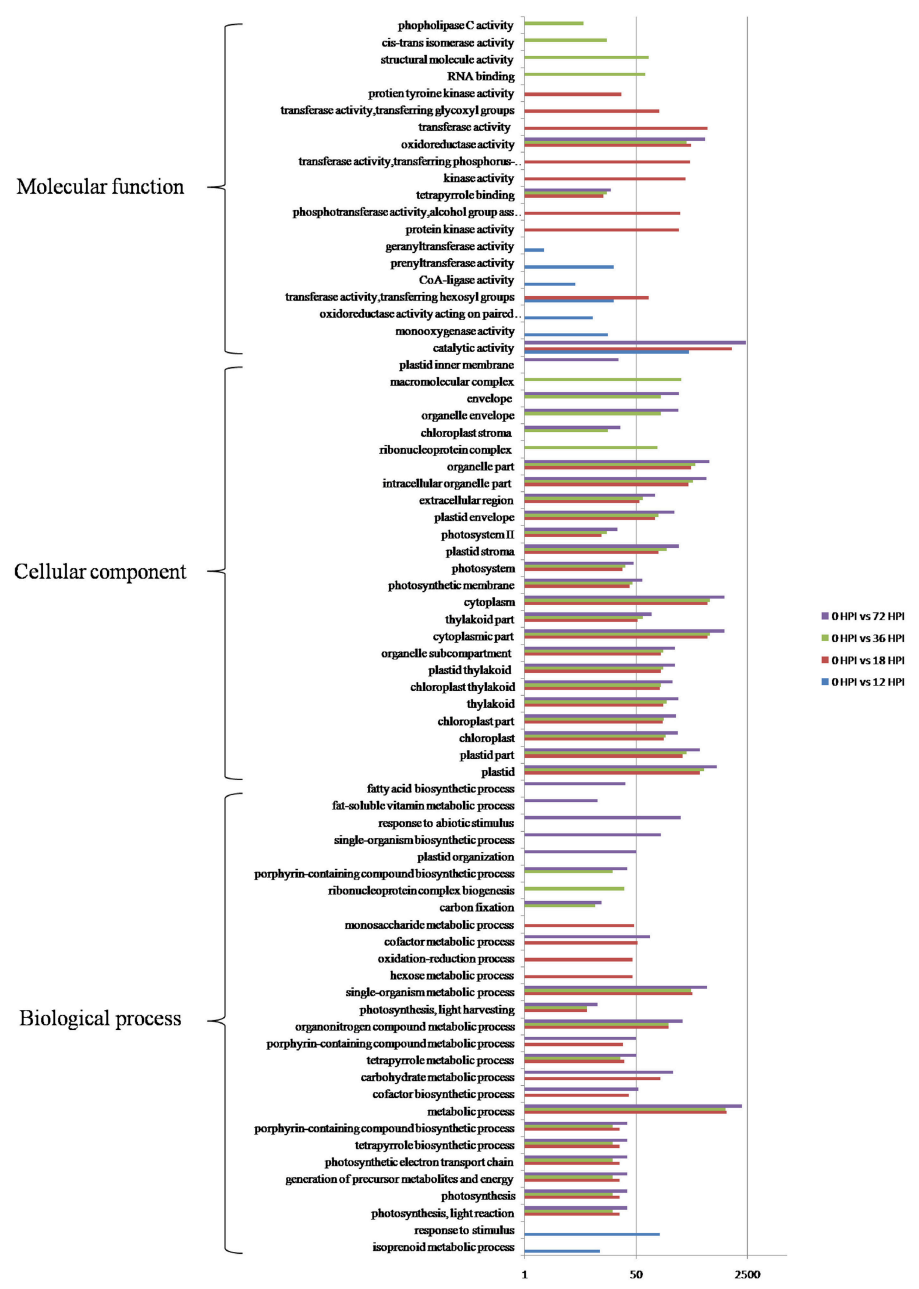
**GO functional enrichment analyzes of DEGs**.

GO categories were developed using the Blast2GO program (http://www.blast2go.com/) in order to evaluate potential DEG functions. DEGs were classified into 50 functional categories including biological process (23), cellular component (15), and molecular function (12) (Figure S1). Results showed that 73 GO terms were enriched as a result of GO enrichment analysis, with the most significant biological process enrichments in the “Metabolic process,” “Single-organism metabolic process,” and “Organonitrogen compound metabolic process.” In contrast, the most significant cellular component enrichments occurred in the “Plastid,” and “Cytoplasmic part and organelle part,” while molecular function enrichments were in “Catalytic activity” and “Oxidoreductase activity.” The results of this analysis also demonstrate that GO terms were enriched differently at each of the four time points. For example, “monooxygenase activity,” “CoA-ligase activity,” and “prenyltransferase activity” as well as “response to stimulus” were particularly enriched by 12 HPI, while “kinase activity,” and “transferase activity” as well as “hexose metabolic process” were particularly enriched by 18 HPI. In addition, “RNA binding,” “structural molecule activity,” and “cis-trans isomerase activity” as well as “ribonucleoprotein complex biogenesis” were particularly enriched by 36 HPI, while “single-organism biosynthetic process,” “plastid organization,” “response to abiotic stimulus,” and “fatty acid biosynthetic process” were particularly enriched by 72 HPI (Figure 3). In addition, KEGG pathway mapping was also carried out based on orthology (KO) terms for assignments; results show that 45 KEGG pathways were significantly enriched. Of these, 17, 30, 22, and 32 pathways were enriched at 12, 18, 36, and 72 HPI, respectively, while nine were enriched at all measured time intervals (Table 1). Maps with highest DEGs representation those for the metabolic pathways (i.e., KO 01100 with 8688 DEGs), followed by those for the biosynthesis of secondary metabolites (i.e., KO 01110 with 5111 DEGs), plant-pathogen interactions (i.e., KO 04626 with 3658 DEGs), and plant hormone signal transduction (i.e., KO 04075 with 2107 DEGs). Taken together, these results suggest that apple have evolved a range of different molecular defense strategies depending on the infection stages of the pathogen.

**Table 1:** **Results of KEGG pathway enrichment analysis**.

**Pathway**	**Pathway ID**	**Number of genes with pathway annotation**	**Number of DEGs at each individual time point**			
			**12 HPI**	**18 HPI**	**36 HPI**	**72 HPI**
Sesquiterpenoid and triterpenoid biosynthesis	Ko00909	130	8			
Synthesis and degradation of ketone bodies	Ko00072	15	3			
Tryptophan metabolism	Ko00380	159	8			
Phenylpropanoid biosynthesis	Ko00940	1057	30			
Terpenoid backbone biosynthesis	Ko00900	186	12	30		
Stilbenoid, diarylheptanoid, and gingerol biosynthesis	Ko00945	567	23	58		
Plant hormone signal transduction	Ko04075	2107	53	203		
Carotenoid biosynthesis	Ko00906	337	16	45		66
Circadian rhythm-plant	Ko04712	294	35	60	45	78
Biosynthesis of secondary metabolites	Ko01110	5111	147	557	449	825
Flavonoid biosynthesis	Ko00941	666	34	92	81	121
Cyanoamino acid metabolism	Ko00460	396	20	63	55	86
Limonene and pinene degradation	Ko00903	338	17	44	36	58
Metabolic pathways	Ko01100	8688	182	786	721	1299
Galactose metabolism	Ko00052	226	11	30	27	47
ABC transporters	Ko02010	358	14	64	42	76
Flavone and flavonol biosynthesis	Ko00944	306	12	36	34	53
Riboflavin metabolism	Ko00740	57		13		
Benzoxazinoid biosynthesis	Ko00402	184		26		
Phenylalanine, tyrosine, and tryptophan biosynthesis	Ko00400	166		24		
Vitamin B6 metabolism	Ko00750	69		12		
Glycolysis/Gluconeogenesis	Ko00010	537		55		
Pentose phosphate pathway	Ko00030	165		28		36
Glycine, serine, and threonine metabolism	Ko00260	241		32		51
Fructose and mannose metabolism	Ko00051	241		30		51
Linoleic acid metabolism	Ko00591	80		13		24
Isoquinoline alkaloid biosynthesis	Ko00950	97		14		24
Photosynthesis-antenna proteins	Ko00196	63		24	28	30
Photosynthesis	Ko00195	175		42	47	67
Glyoxylate and dicarboxylate metabolism	Ko00630	174		34	25	36
Porphyrin and chlorophyll metabolism	Ko00860	213		37	36	63
Carbon fixation in photosynthetic organisms	Ko00710	304		44	37	65
Ascorbate and aldarate metabolism	Ko00053	232		30	34	60
Amino sugar and nucleotide sugar metabolism	Ko00520	453		49	50	79
Ribosome	Ko03010	968			107	
Ribosome biogenesis in eukaryotes	Ko03008	409			47	
Plant-pathogen interaction	Ko04626	3658			193	489
Nitrogen metabolism	Ko00910	166			23	38
Propanoate metabolism	Ko00640	150			21	32
Starch and sucrose metabolism	Ko00500	920			84	152
Inositol phosphate metabolism	Ko00562	183				36
Ubiquinone and other terpenoid-quinone biosynthesis	Ko00130	128				30
Histidine metabolism	Ko00340	82				14
Valine, leucine, and isoleucine degradation	Ko00280	155				31
Lysine degradation	Ko00310	91				20

### Validation of RNA-Seq expression levels by qRT-PCR

**Figure 4:**
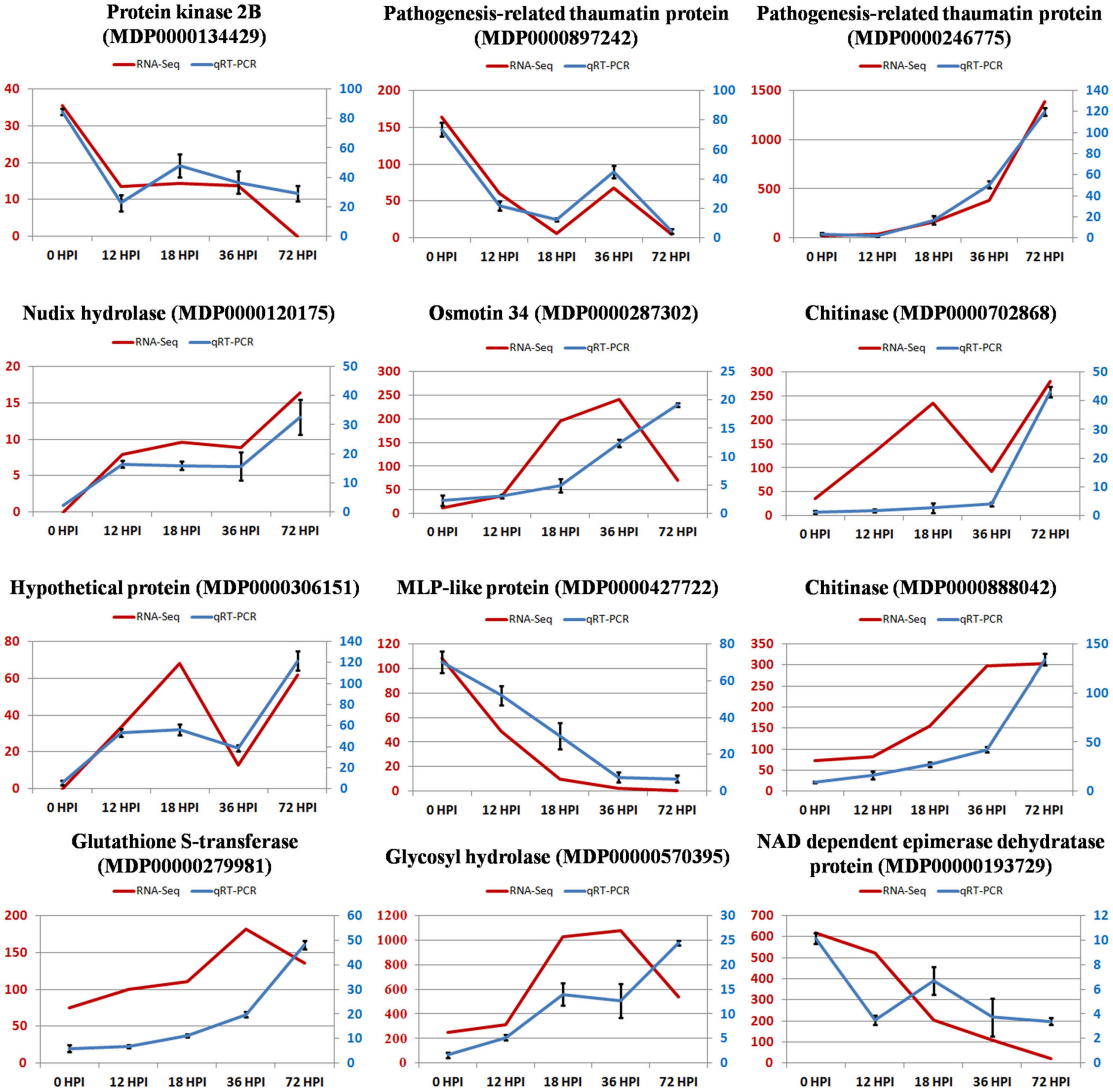
**The relative expression level change of 12 selected genes from DEGs by quantitative real-time PCR**. Left vertical coordinate is RPKM of RNA-Seq; right vertical coordinate is relative expression level of qRT-PCR.

Total RNA at four time points during the pathogen infection provided templates for qRT-PCR validation. We randomly selected 12 DEGs to validate our RNA-Seq results. qRT-PCR data for these genes were consistent with the RNA-Seq results from the four samples, which indicated a high degree of reproducibility between transcript abundances assayed using RNA-Seq and the expression profiles revealed by qRT-PCR data (Figure 4). However, the fact that quantities were not highly consistent suggests sensitivity differences between the two methods.

### DEGs involved in cell wall reinforcement or disassembly

**Figure 5:**
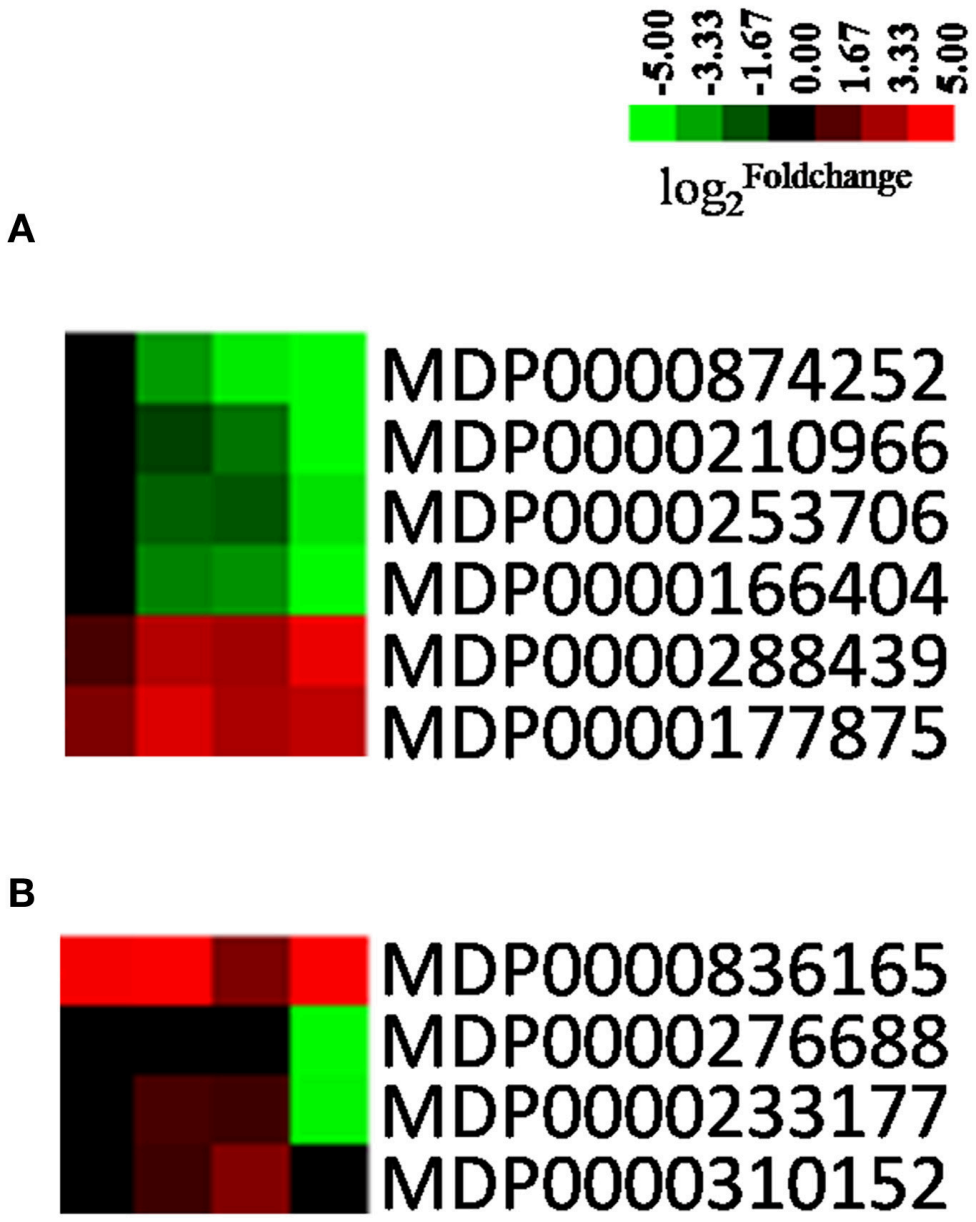
**Heatmaps of DEGs involved in cell wall reinforcement or disassembly**. The $\log_{2}^{\left|\text{Foldchange}\right|}$ was colored using Cluster 3.0 (i.e., red for up-regulated, green for down-regulated), each horizontal row represents a DEG with its gene ID, and the vertical columns represent 12, 18, 36, and 72 HPI from left to right. **(A)** Genes related with cuticle. **(B)** Genes related to cell walls.

Plant cell wall forms a dynamic physical barrier that protects cell from microbial infection. Prospective plant pathogens must overcome the physical barrier presented by cuticle and plant cell wall. The cuticle is the first cell wall layer encountered by a pathogen, and its biosynthesis involves a number of genes, including *WSD* (*wax-ester synthase/diacylglycerol O-acyltransferase*; Li et al., 2008), *CYP77A6* (*cytochrome P450*; Yeats et al., 2010), *CYP94A1* (*fatty acid omega-hydroxylase*; Pinot et al., 1999; Pinot and Beisson, 2011), and *HTH* (*HOTHEAD, fatty acid omega-hydroxy dehydrogenase*; Kurdyukov et al., 2006). Our results show that the homologs of these genes in apple, *WSD* (*MDP0000253706*), *HTH* (*MDP0000210966*), *CYP77A6* (*MDP0000874252*), and *CYP94A1* (*MDP0000166404*), were all down-regulated during the infection process (Figure 5A), which implies that the biosynthesis of cutin, suberine, and wax was impaired in infected leaves making it easier for AAAP to penetrate. We also speculate that the weakness of this physical barrier may be one of the reasons why “Starking Delicious” apples are so susceptible to AAAP.

Fungal necrotrophs degrade the cuticle via the action of cutinases and lipases (Bellincampi et al., 2015). These cuticular breakdown products can be sensed by plants, initiating activation of a series of genes associated with cell wall reinforcement and modification (Schweizer et al., 1996). Calloses, produced by callose synthase (CALS), are thought to reinforce the cell wall at sites of fungal penetration in an effort to impede infections (Underwood, 2012), while *PME* (*pectin methylesterase*), *XTH* (*xyloglucan endotransglycosylase/hydrolase*) are both genes that encode enzymes involved in cell wall degradation or modification (Albert et al., 2004; Raiola et al., 2011). The results of this study demonstrate that *CALS1* (*MDP0000177875*) is up-regulated while *PME* (*MDP0000276688*) and *XTH* (*MDP0000233177*) are down-regulated, suggesting that apples are able to sense the signals of pathogen infection and activated relevant defense mechanisms. Nevertheless, we also found that paralogous genes of *PME* (*MDP0000276688*) and *XTH* (*MDP0000233177*) were up-regulated in the early stages of infection (Figure 5B); in *Arabidopsis*, the *AtPME3* acts as a susceptibility factor, required by necrotrophic pathogens for the initial colonization of host tissue (Raiola et al., 2011). We hypothesize that the up-regulation of *PME* in the early stages of infection might provide the conditions for the initial colonization of apple leaves by AAAP.

### DEGs involved in phytohormone signaling

**Figure 6:**
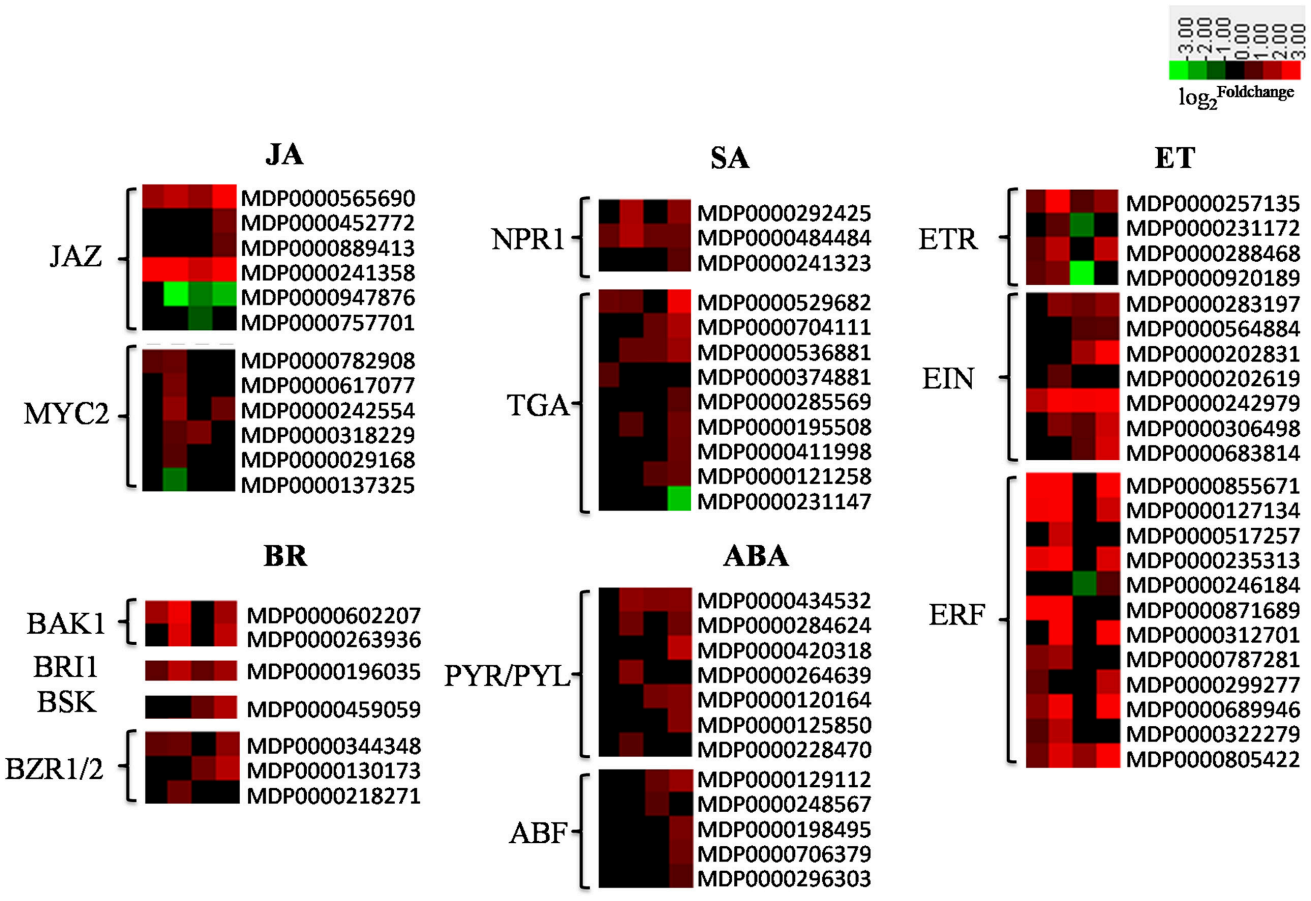
**Heatmaps of DEGs involved in phytohormone signaling pathways, including JA, SA, ET, BR, and ABA signaling pathways**. The $\log_{2}^{\left|\text{Foldchange}\right|}$ was colored using Cluster 3.0 (red for up-regulated, green for down-regulated), each horizontal row represents a DEG with its gene ID, and the vertical columns represent 12, 18, 36, and 72 HPI from left to right. Abbreviations for genes on the left of the heatmap are defined in the text.

Phytohormones, including salicylic acid (SA), ethylene (ET), brassinosteroids (BR), abscisic acid (ABA), and jasmonic acid (JA), are critical regulators of plant-pathogen interactions (De Vleesschauwer et al., 2013). In the experiment, most of the genes involved ET (e.g., *ERF, EIN*, and *ETR*) and SA signaling (e.g., *NPR1* and *TGA*) were up-regulated when the “Starking Delicious” cultivar was challenged by AAAP, while differential expression of the NPR1 gene, the key regulator of SA-mediated transcriptional reprogramming and immunity (Pajerowska-Mukhtar et al., 2013), was up-regulated more than four-fold (Figure 6, Excel S3). Traditionally it has been thought that SA mediates defense signaling against biotrophic and hemibiotrophic pathogens, while JA and ET are associated with defense responses to necrotrophs (Kunkel and Brooks, 2002; Glazebrook, 2005). Our results suggest that SA might also play a role in local immunity against necrotrophic pathogens.

It has also been shown that ABA is a crucial regulator of plant-microbe interactions (Asselbergh et al., 2008; Cao et al., 2011). It has been repeatedly shown to paralyze plant defenses by antagonizing the SA pathway in *Arabidopsis*, thus it predominantly behaves as a negative regulator of immunity (Koga et al., 2004; De Vleesschauwer et al., 2013; Xu et al., 2013). Indeed, in our experiments, the genes involved in ABA signal perception and transduction pathways, for example *PYR/PYL* (*pyrabactin resistance1/PYR1-like*), *ABF* (*ABA binding factor*) were all up-regulated (Figure 6, Excel S3). Finally, BRs comprise a unique class of growth-promoting steroid hormones that have also been shown to be key regulators of plant immunity (Wang, 2012). Intensive research has deciphered the complex positive and negative roles of BRs and their signaling in innate immunity, the genes encoding BR signaling cascades, including *BAK1* (*BRI-associated receptor kinase 1*; Chinchilla et al., 2009; Choudhary et al., 2012), *BRI1* (*brassinosteroid insensitive1*), *BSK* (*brassinosteroid signaling kinases*), and *BZR* (*brassinazole-resistant transcription factor*), were up-regulated as a result of “Starking Delicious” apple in response to AAAP infection (Figure 5, Excel S3).

Interestingly, in our experiments, most of the genes involved in ET and BR biosynthesis and signaling cascades were differentially expressed by 12 HPI, while those involved in JA, SA, and ABA biosynthesis and signaling were not differentially expressed until 18 HPI. These results therefore indicate that ET and BR signaling cascades were triggered earlier by the pathogen than the JA, SA, and ABA signaling cascades.

The involvement and characteristics of differential expression of multiple phytohormone signaling genes suggests that these signals are not just simple linear and isolated cascades, but that they “crosstalk” with each other. Thus, overlaps in defense and the differences in efficacy could also be triggered by divergent pathways at the different stages of AAAP infection.

### Transcription factors (TFs)

**Figure 7:**
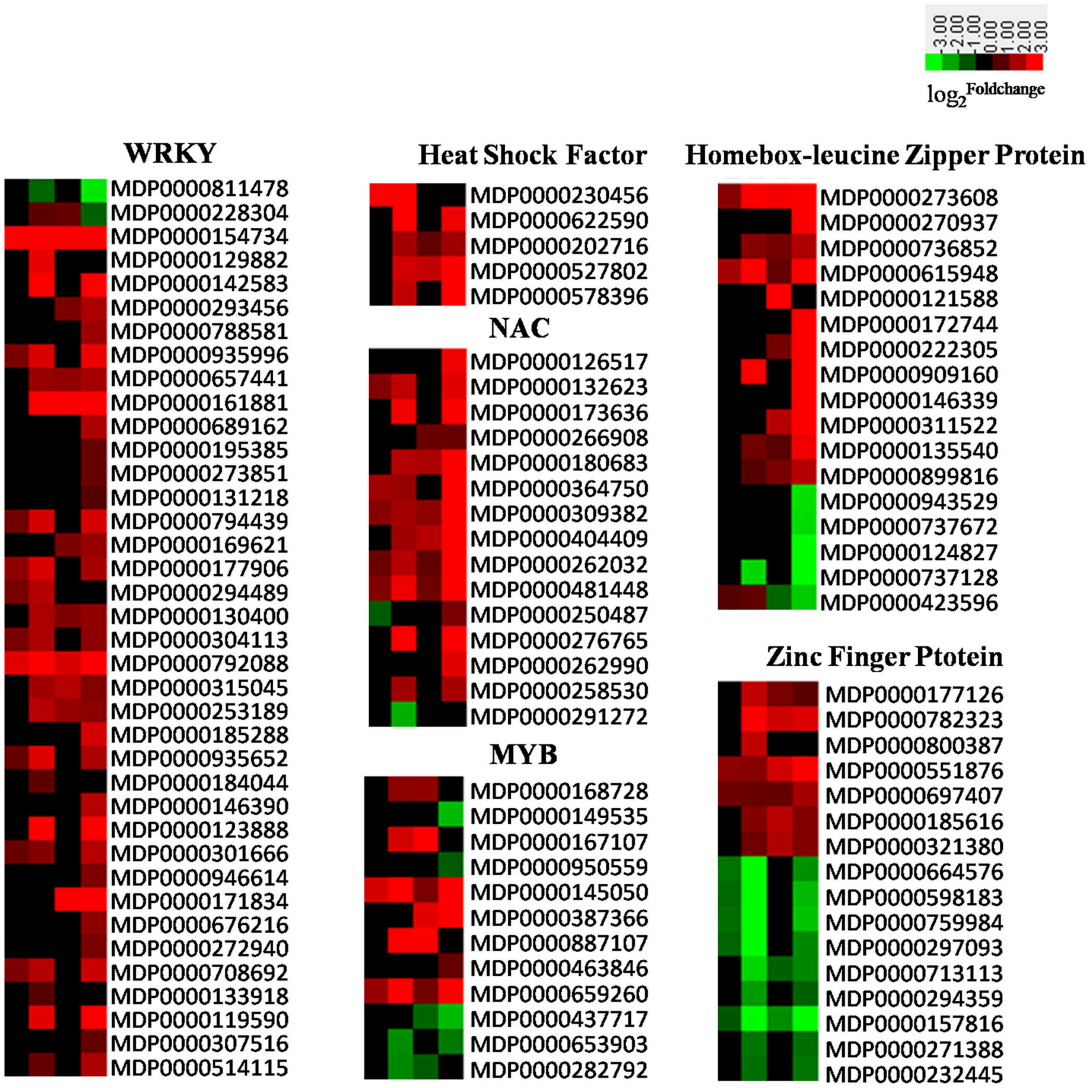
**Heatmaps of DEGs encoding transcriptional factors including WRKYs, HSFs, NACs, MYBs, zinc finger proteins, and homebox-leucine zipper proteins**. The $\log_{2}^{\left|\text{Foldchange}\right|}$ was colored using Cluster 3.0 (i.e., red for up-regulated, green for down-regulated), each horizontal row represents a DEG with its gene ID, and the vertical columns represent 12, 18, 36, and 72 HPI from left to right.

TFs are key regulatory proteins, essential for the regulation of gene expression. In plants, *WRKY* (Pandey and Somssich, 2009), *Hsf* (Pajerowska-Mukhtar et al., 2012), zinc finger protein (Maldonado-Bonilla et al., 2013), *ZIP* (Dezar et al., 2011), and *NAC* (Sun et al., 2013) are all important regulators of plant defense responses. The results of our experiment show that almost all *WRKYs, ZIPs, Hsfs*, and *NACs*, as well as most *MYBs* and a portion of the genes encoding for zinc finger proteins were significantly up-regulated. The expression of related TFs in apple induced by AAAP can be seen in Figure 7 and Excel S3.

There are more than 100 WRKY family-members within the apple genome (http://planttfdb.cbi.pku.edu.cn/family.php?sp=Mdo&fam=WRKY), 38 of which were induced by AAAP infection on the basis of our experiments. Results show that most were up regulated, with the exception of *MDP0000811478* and *MDP0000228304*; indeed, some WRKY genes were significantly up-regulated throughout the whole experimental period, for example *MDP0000154734, MDP0000792088*, and *MDP0000161881*. In particular, *MDP0000154734*, which shows high similarity with *AtWRKY75*, were up-regulated almost 30-fold by 12 HPI, increasing to 180-fold by 72 HPI (Figure 7). Previously work has shown that in *Arabidopsis, AtWRKY75* acts as a positive regulator of JA- or SA-mediated defense signaling responses to the necrotrophic pathogen *Pectobacterium carotovorum* ssp. *carotovorum* (Choi et al., 2014). These results also suggest that the gene homologous to *AtWRKY75* in apple may also play an important role in responses to AAAP infection.

An increasing body of evidence has implicated NAC genes in the defense responses of plant to pathogen infections and environmental stimuli (Xia et al., 2010; Nuruzzaman et al., 2015). It is thought that NAC genes positively regulate plant defense responses by activating PR genes, inducing a hypersensitive response (HR) and cell death at the site of infection, or negatively regulate plant basal pathogen resistance by suppressing *PR1* expression (Jensen et al., 2007; Kaneda et al., 2009; Seo et al., 2010; Kim et al., 2012). In our experiment, 15 *NAC* genes exhibited differential expression out of a total of 180 in the apple genome (Figure 7; Su et al., 2013). Results show that most of these were up-regulated, with the exception of *MDP0000291272* and *MDP0000250487*, while in *Arabidopsis* over-expression of *ATAF2*, a NAC transcription factor gene, which has a high degree of similarity with *MDP0000291272*, resulted in the repression of a number of PR proteins and higher susceptibility to the soil-borne fungal pathogen *fusarium oxysporum* (Delessert et al., 2005). We therefore hypothesize that up-regulation of most NAC genes combined with down-regulation of gene *MDP0000291272* in the early stage of infection may play a positive role in the defense of the “Starking Delicious” apple cultivar against AAAP.

The MYB gene family is extraordinarily large in apple (Cao et al., 2013). These genes are involved in a variety of functions, including the anthocyanin biosynthetic pathway (Espley et al., 2007), morphogenesis (Vimolmangkang et al., 2013), and abiotic stress responses (Wang et al., 2014). There have been relatively few reports dealing with the responses of MYBs in biotic stress, although it is known that the R2R3 MYB transcription factor in wheat mediates host resistance to the *Bipolaris sorokiniana* pathogen via regulation of SA-signaling pathways and defense-related genes (Zhang et al., 2012). A total of 12 apple MYB genes were either up- or down- regulated by AAAP infection in our experiment (Figure 7), which suggests that these genes might also play a regulatory role in responses of the “Starking Delicious” cultivar against AAAP attack. However, this hypothesis requires confirmation by additional research.

Heat shock transcription factors (Hsfs) are also thought to be involved in a range of pathological conditions, cellular responses to oxidative stress, as well as certain developmental and differentiation processes (Morimoto, 1998; Hahn et al., 2004). There are 25 members of the Hsf family in apple genome (Giorno et al., 2012), of which five were induced by AAAP infection and were up-regulated in our experiments (Figure 7). Three of these can be classified as class B-Hsf, and *MDP0000527802* and *MDP00006225590* in particular significantly increased in expression following AAAP infection. These two genes exhibit a high degree of similarity with *AtHsfB2b* and *AtHsfB1*, respectively, while the double knockout *hsfb1 and hsfb2b* genes in *Arabidopsis* significantly improved disease resistance following *A. brassicicola* infection via strong up-regulation of the basal mRNA-levels in the defensin genes *Pdf1.2a/b* (Kumar et al., 2009). We therefore speculate that the up-regulation of class B-Hsfs genes in apple is counterproductive to disease resistance.

In addition, results show that a number of homeobox-leucine zipper proteins and zinc finger proteins were also induced by AAAP infection (Figure 7). However, unlike the other TFs discussed above, most zinc finger proteins were down-regulated in our experiments.

### Defense-related proteins

**Figure 8:**
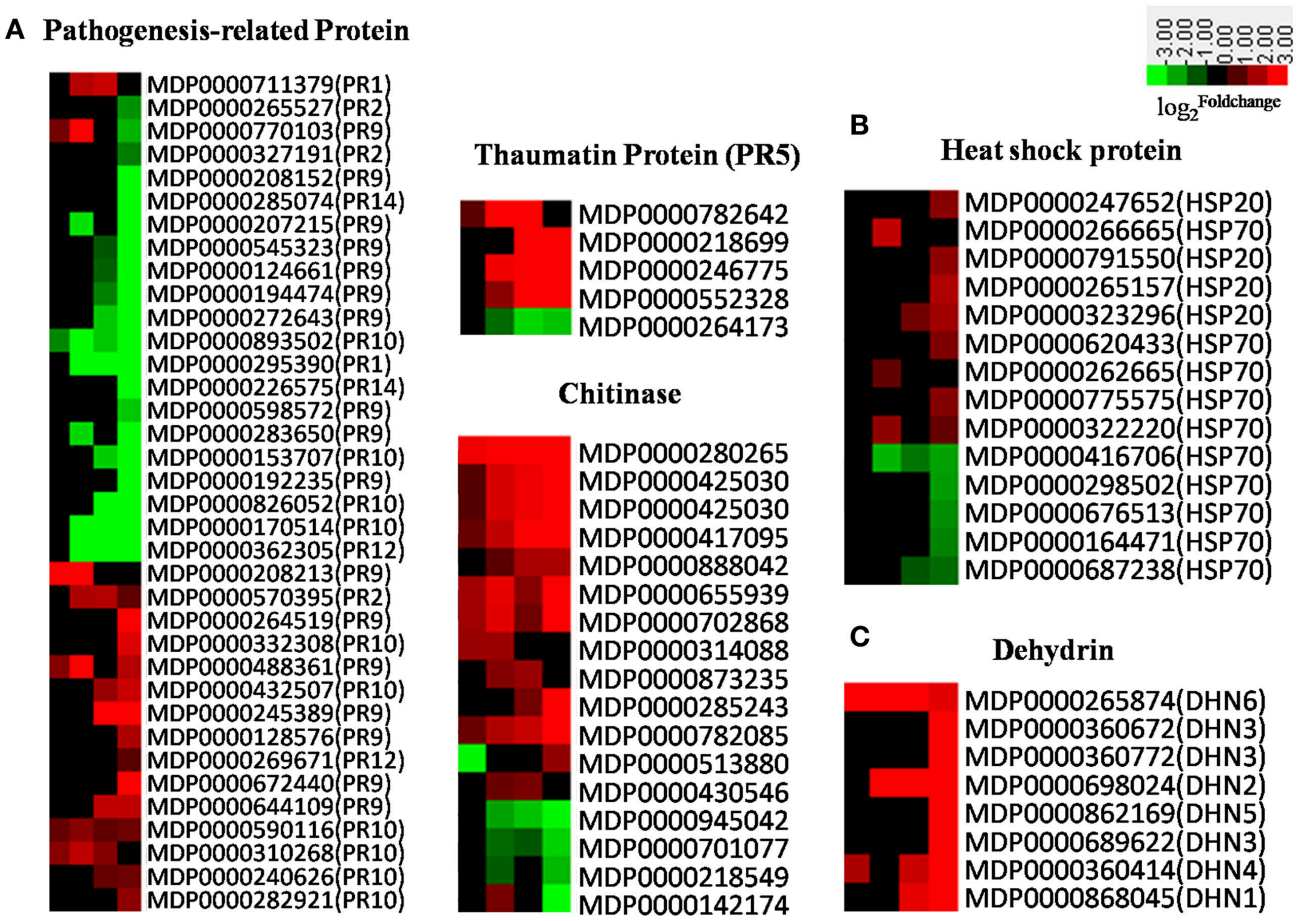
**(A)** Heatmaps of DEGs encoding pathogenesis-related (PR) proteins; **(B)** Heatmap of DEGs encoding heat shock proteins; **(C)** Heatmap of DEGs encoding dehydrin. The $\log_{2}^{\left|\text{Foldchange}\right|}$ was colored using Cluster 3.0 (i.e., red for up-regulated, green for down-regulated), each horizontal row represents a DEG with its gene ID, and the vertical columns represent 12, 18, 36, and 72 HPI from left to right.

There are 17 families of PR proteins, normally expressed at nearly undetectable levels in healthy tissues, but which rapidly accumulate to significant amounts in response to biotic or abiotic stress (Van Loon and Van Strien, 1999; van Loon et al., 2006). The accumulation of PR proteins is usually associated with systemic acquired resistance to a wide range of pathogens (Ward et al., 1991; Durrant and Dong, 2004). Apple PR genes were induced in response to AAAP infection in our experiment (Figure 8A); the expression of PR-1, most PR-2s, PR thaumatin-like proteins (PR-5s), and defense-regulated genes were all associated with SA-regulated defense responses (Ward et al., 1991; Yang et al., 1997) and gradually increased subsequent to 18 HPI (Figure 8A). Results also show similar expression patterns in SA receptor genes, *NPR1s* and *TGAs* (Figure 6), while *chitinases*, JA-regulated defense genes, responded more quickly to AAAP infection than SA-regulated counterparts. Our results thus demonstrate that JA signal transduction is dominant at early infection stages, and that most other PR genes, including *PR-9s* (peroxidases), *PR-10s* (ribonucleases), *PR-12s* (defensins), and *PR-14s* (lipid-transfer proteins), were down-regulated subsequent to 18 HPI. Overall, PR genes, with the exception of *chitinases*, fail to respond to AAAP infection timely in the “Starking Delicious” apple cultivar. It was known that the timing of PR gene expression was a crucial determinant of pathogenesis: Response at an early stage of infection was favorable to plant prevention of violation by the pathogen (Kaur et al., 2011). Down-regulation and lagged expression of most *PR* genes might imply that the “Starking Delicious” apple cultivar is sensitive to AAAP infection.

Heat shock proteins (HSPs) are a subset of molecular chaperones, best known because they are rapidly induced in large numbers by stress (Neumann et al., 1994; Wang et al., 2004; Scarpeci et al., 2008). These proteins act as molecular chaperones to stabilize, reduce misfolding, and facilitate the refolding of proteins denatured as a result of stresses. Our results show that *HSPs* in the “Starking Delicious” cultivar exhibited a range of responses to AAAP. *HSP20s*, for example, did not respond at early stages of infection but slightly increased at later stages. In contrast, many of HSP70 transcripts were down-regulated (Figure 8B), a result confirmed by a proteomics study of apple-AAAP interactions that showed that these proteins accumulate in resistance as opposed to susceptible leaves (Zhang et al., 2015). Additional research has shown that HSP70s are induced by both *P. expansum* and *P. digitatum* pathogens in “Ultima Gala” apple cultivar (Spadoni et al., 2015), thus expression of these proteins is an important fact of apple disease-resistance. Down-regulation of *HSP70s* in the apple leaves suggests that the defense signal transduction pathway mediated by HSP70s may be compromised in the “Starking Delicious” cultivar, leading to AAAP susceptibility.

Apple dehydrins (DHNs) are another family of proteins that protect cells from damage caused by a variety of abiotic stresses, including drought, salt, and low temperature (Liang et al., 2012). A number of studies have confirmed that DHNs are also involved in biotic stress; for example, over-expression of wheat *DHN5* in *Arabidopsis* induced the up-regulation of PR genes and led to increased resistance to fungal infections caused by *Botrytis cinerea* and *Alternaria solani* (Brini et al., 2011; Drira et al., 2016). Other research has shown that the grapevine DHN1 protein was up-regulated in *Vitis yeshanensis* in response to *Eryiphe necator* infection (Yang et al., 2012), while in our experiments, DHN genes in apple, including *DHN1-6*, were up-regulated following AAAP infection, with *DHN4* (*MDP0000360414*) and *DHN6* (*MDP0000265874*) responding particularly rapidly (Figure 8C). These results suggest that *DHNs* may play a protective role when plants respond to biotic stresses.

### DEGs involved in photosynthesis, oxidation-reduction, amino acid, and secondary metabolism

**Figure 9:**
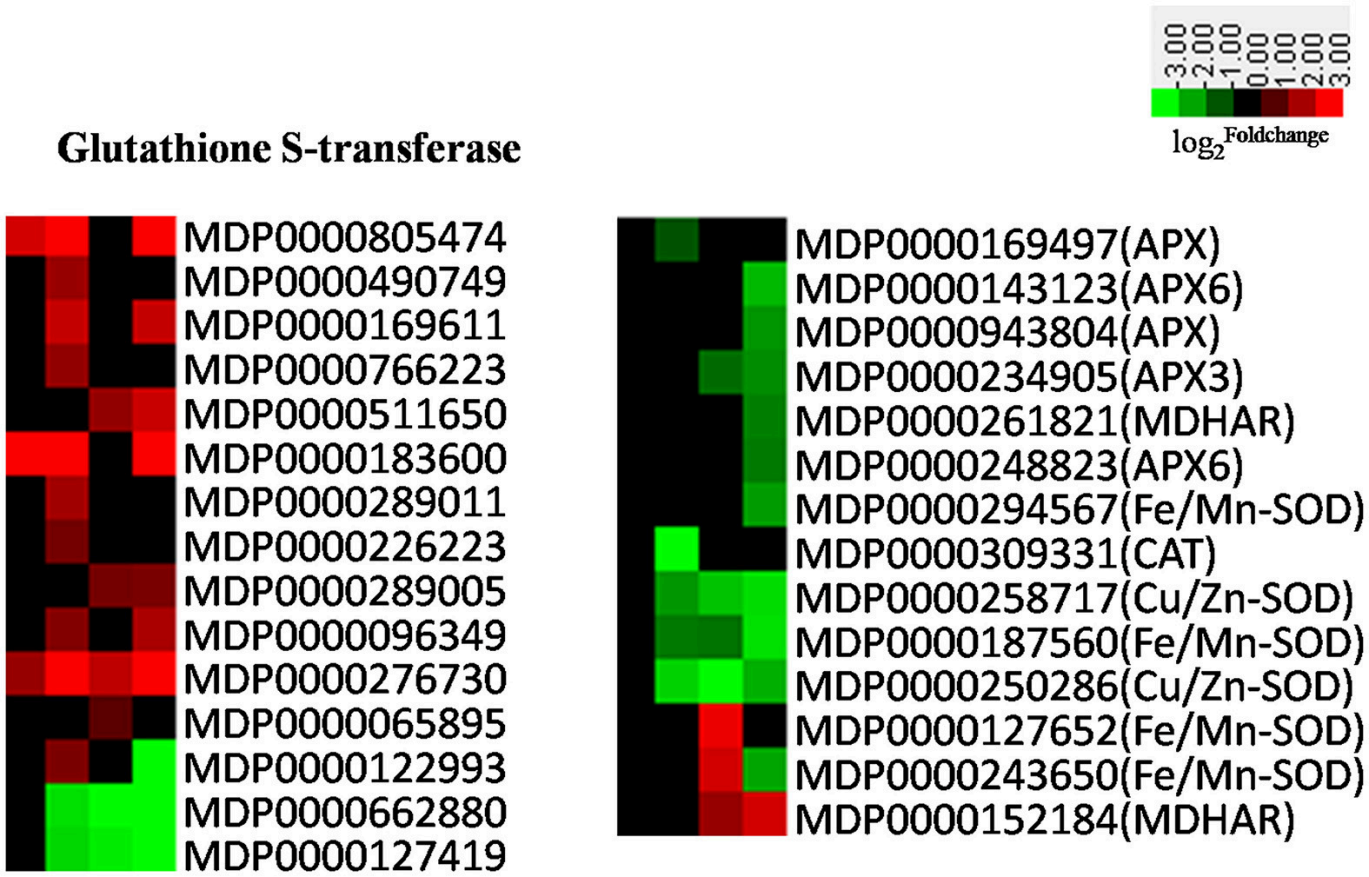
**Heatmaps of DEGs involved oxidation-reduction**. The $\log_{2}^{\left|\text{Foldchange}\right|}$ was colored using Cluster 3.0 (i.e., red for up-regulated, green for down-regulated), each horizontal row represents a DEG with its gene ID, and the vertical columns represent 12, 18, 36, and 72 HPI from left to right.

Our results confirm that almost all DEGs involved in photosynthesis were down-regulated as a result of pathogen infection (Figure S2). This indicates that the rate of photosynthesis gradually declined as disease progressed. Hundreds of transcripts associated with amino acid metabolism were differentially expressed in apple leaves attacked by AAAP, with some very significantly activated in later infection stages on the basis of KEGG pathway enrichment analysis (Table 1). Our results also demonstrated that a decrease in photosynthesis can be attributed to plants adjusting their metabolism to produce defense-related compounds (Walters and Boyle, 2005). Results also demonstrate that the genes encoding glutathione-S-transferases (GSTs), superoxide dismutases (SODs), catalases (CATs), L-ascorbate peroxidases (APXs), and monodehydroascorbate reductases (MDHARs), which all involved in the regulation of redox homeostasis, were all down-regulated as the result of AAAP infection (Figure 9).

**Figure 10:**
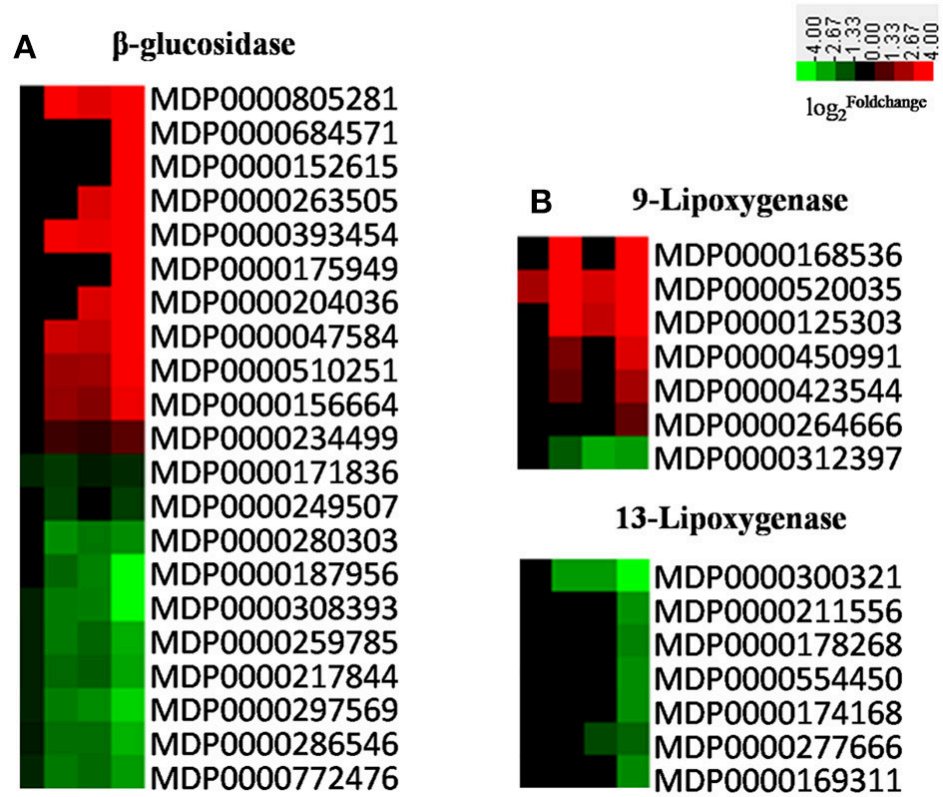
**Heatmaps of differentially expressed (A)** *BGLs* and **(B)** *LOXs*. The $\log_{2}^{\left|\text{Foldchange}\right|}$ was colored using Cluster 3.0 (i.e., red for up-regulated, green for down-regulated), each horizontal row represents a DEG with its gene ID, and the vertical columns represent 12, 18, 36, and 72 HPI from left to right.

Secondary metabolites function in biocidal defense and as regulatory signals in innate immunity (Dixon, 2001; Grayer and Kokubun, 2001). The differential expression of many genes encoding key enzymes in the lignin, phenylpropanoid, flavonoids, benzoxazinoids, cyanoanimo acid, alkaloid, and terpene biosynthetic pathways were detected in our experiment (Figure S3), of which β-glucosidases (BGLs) play an important defensive role by releasing glucosyl-blocking groups from metabolic intermediates and allowing for the modification of various phytoanticipins and phytotoxins (Morant et al., 2008; Cairns and Esen, 2010). The expression level of *BGLs* were drastically up- and down-regulated in our experiments (Figure 10A), and the presence of differentially-expressed isozyme genes suggested that these proteins have a range of different functions in apple in response to biotic stresses. In addition, oxylipins act as protective compounds and are potential signaling molecules (Blée, 2002), and lipoxygenases (LOXs) are key enzymes mediating their synthesis. The results of this study show that the majority of *9-LOXs* were up-regulated in AAAP-infected leaves, while no difference in expression level was seen in the majority of *13-LOXs* up to 72 HPI and the expression levels of *13-LOXs* were then subsequently down-regulated by 72 HPI (Figure 10B). A body of research has demonstrated that the 9-LOX pathway is essential to plant defense against microbial pathogens (Rancé et al., 1998), and that 13-LOX-derived oxylipin biosynthesis is associated with HR induction in *Arabidopsis* during race-specific resistance to the *Pseudomonas syringae* pathogen (Veronico et al., 2006). Moreover, the first step of JA biosynthesis is catalyzed by a member of the 13-LOX family (Bannenberg et al., 2009). Therefore, we speculate that down-regulated expressions of these genes may impair the defense response of apple leaves.

## Conclusions

In conclusion, transcriptomic analysis allowed us to detect a total of 9080 DEGs in the “Starking Delicious” apple cultivar in response to AAAP infection. GO and pathway enrichment analysis indicated that these DEGs were predominately involved in both biological processes and metabolic pathways. Almost all genes associated with photosynthesis and oxidation-reduction were down-regulated, and a large number of defense-related genes and transcription factors were activated as a result of the infection process. Transcriptomic data suggested that cell wall defensive vulnerability and the down-regulation of most PRs and HSP70s genes in the “Starking Delicious” cultivar following infection may present possible reasons for susceptibility to AAAP.

## Author contributions

SW was the recipient of funds; SW, LZ, and HX conceived the experiment. SL and BC prepared the plant materials and collected samples. LZ, WN, and BC undertook experiments and data analysis. SW, LZ, and HX prepared the manuscript.

### Conflict of interest statement

The authors declare that the research was conducted in the absence of any commercial or financial relationships that could be construed as a potential conflict of interest.
