# Supplementary material for: Transcriptomics Analysis of Apple Leaves in Response to Alternaria alternata Apple Pathotype Infection
Source: Front Plant Sci. 2017 Jan 20;8:22. doi: 10.3389/fpls.2017.00022 (PMC5248534; doi:10.3389/fpls.2017.00022)
Supplement: Supplementary file 1 [file DataSheet1.ZIP › Data sheet 1/Supplementary Material.docx]

***Supplementary materials***

**Transcriptomics analysis of apple leaves in response to *Alternaria alternata* apple pathotype infecton**

**Longming Zhu^1, 3^, Weichen Ni^1^, Shuai Liu^1^, Zongming Cheng^1, 2^, Binhua Cai^1^, Han Xing^3^, Sanhong Wang^1*^**

* Correspondence:

Sanhong Wang

E-mail: wsh3xg@hotmail.com

**1 Supplementary Data**

Supplementary Excel S1. All detected genes

Supplementary Excel S2. Up-regulated and down-regulated genes

Supplementary Excel S3. Selected a representative of the differentially expressed genes

**2** **Supplementary Figures and Tables**

**2.1 Supplementary Figures**


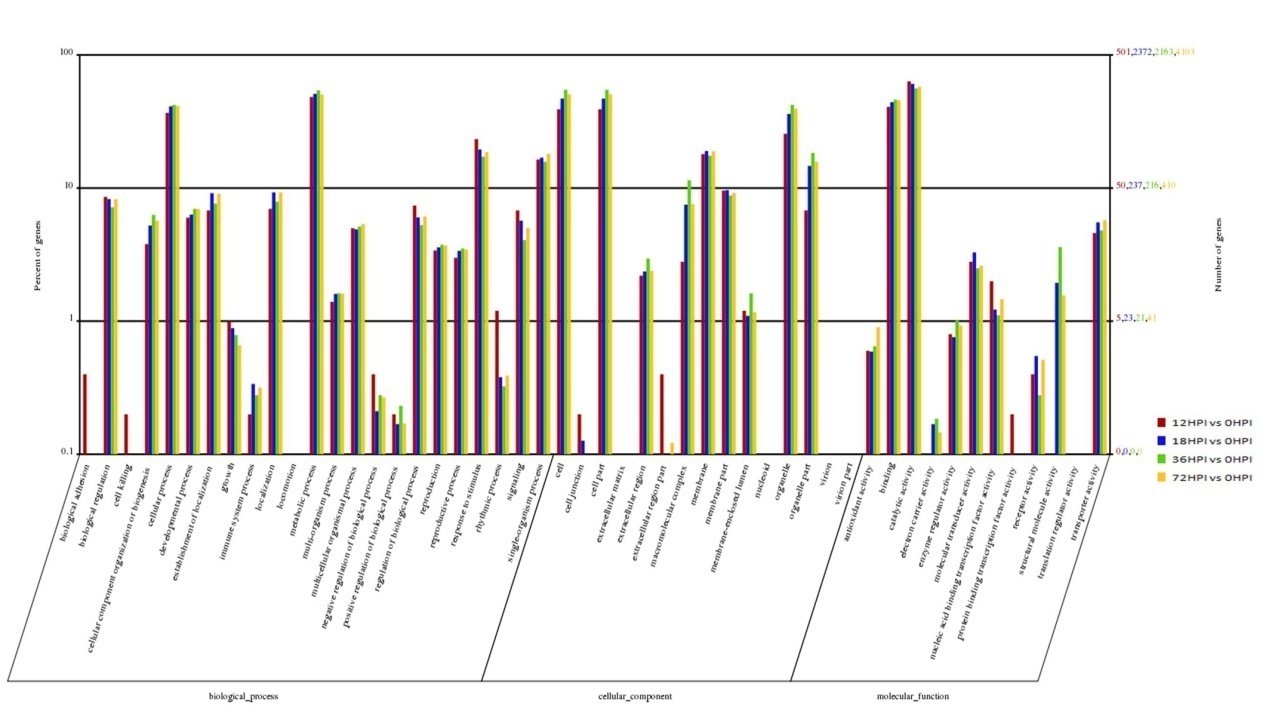


**Supplementary Figure S1.** Gene ontology functional categories of differentially expressed genes.


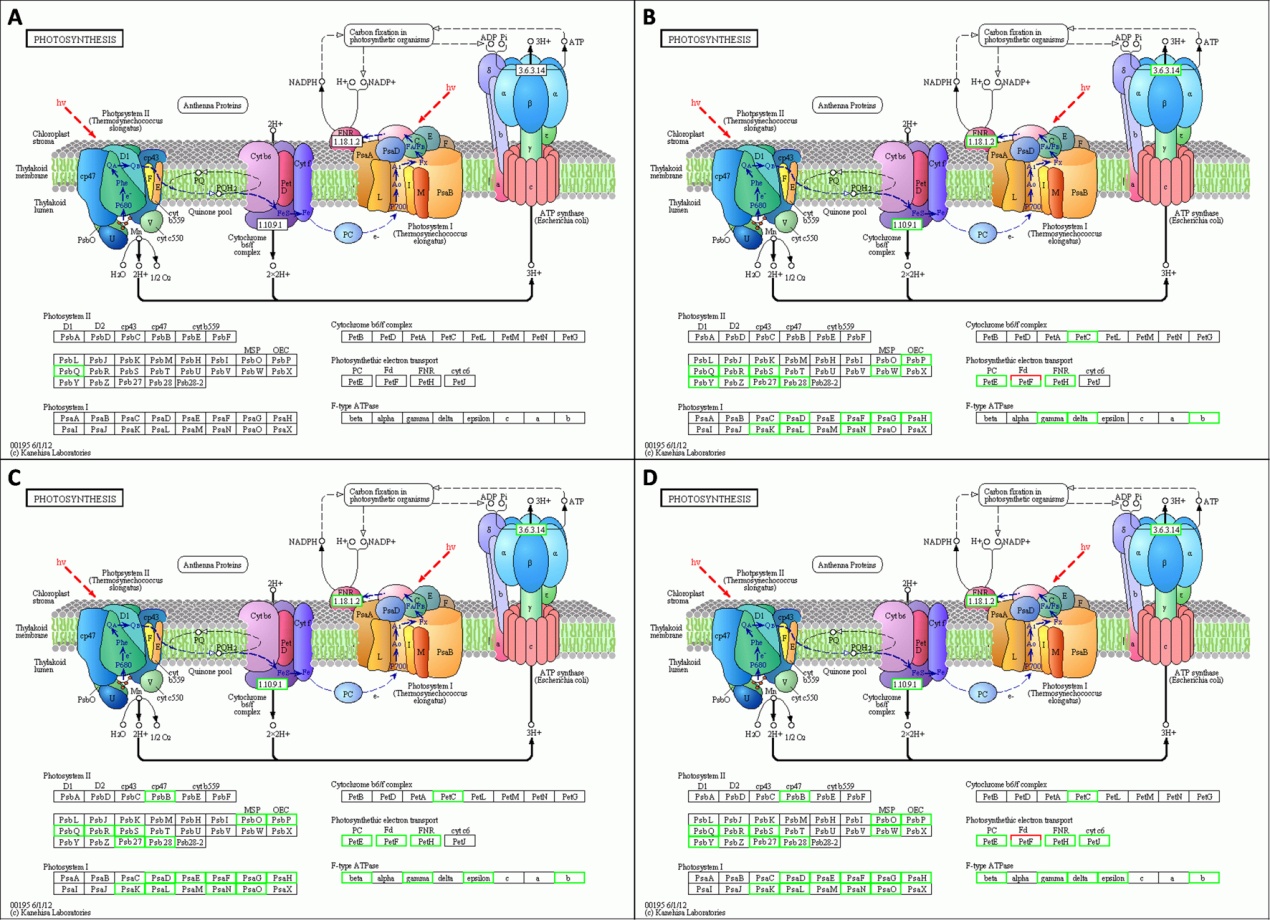


**Supplementary Figure S2.** KEGG pathways of photosynthesis labeled with related differentially expressed genes at different time points: (A) 12 HPI, (B) 18 HPI, (C) 36 HPI, (D) 72 HPI.


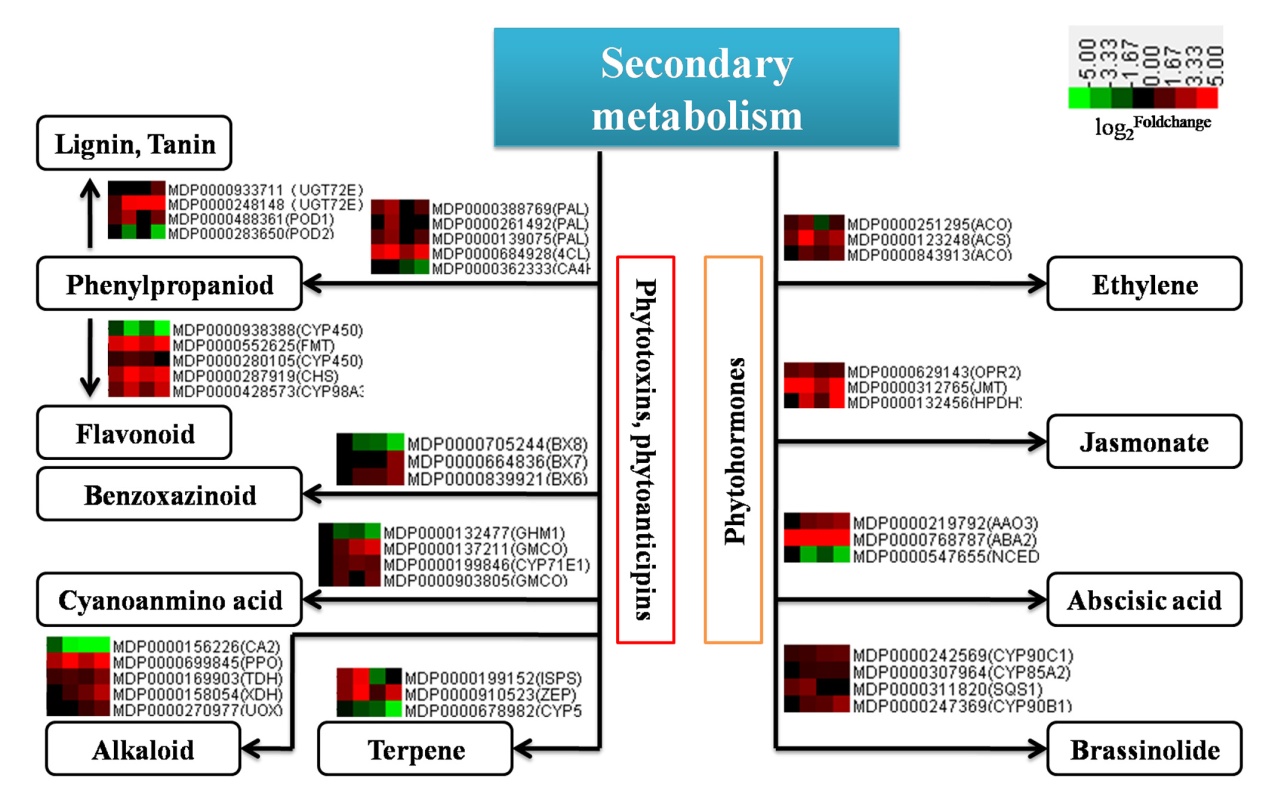


**Supplementary Figure S3.** A brief view of secondary metabolism decorated with the heatmap of differentially expressed genes encoding key enzymes in secondary metabolism. The log_2_^Foldchange^ were colored by Cluster 3.0 (red for up-regulated, green for down regulated). Each horizontal row represents a differentially expressed gene with its gene ID, and the vertical columns represent 12, 18, 36 and 72 HPI from left to right. Information of the differentially expressed genes can be found in supplementary Excel S3.

**2.2 Supplementary Tables**

**Supplementary Table S1. Specific primers used in relative quantitative real-time RT-PCR**

| **Gene name** | **Gene ID** | **Primer sequence (5’ to 3’)** | **Tm (℃)** | **AS(bp)** |
| --- | --- | --- | --- | --- |
| Nudix hydrolase 1 | MDP0000120175 | FP: GAGAAGATAGAGGTGCTGAA  RP: AAGAGTGGTTTGGGTAGATT | 60.2  60.3 | 185 |
| Protein kinase 2B | MDP0000134429 | FP: CGGATTCGGTGATGTCTA  RP: CATTGTTCATCGCCTTGT | 60.0  59.8 | 129 |
| Hypothetical protein | MDP0000306151 | FP: TTACGAACCAACAAACTCTC  RP: CCAGTACCAGGACTTGAG | 59.7  60.1 | 124 |
| Pathogenesis-related thaumatin superfamily protein | MDP0000897242 | FP: ATCCTACACACCCTCTCT  RP: TCCTTGAAACAACTCCTGA | 60.0  60.0 | 165 |
| Osmotin 34 | MDP0000287302 | FP: ACCGTGTTCAAGACTGAT  RP: CAACCTTGTAGTTAGTTCCAG | 60.0  60.0 | 160 |
| MLP-like protein 423 | MDP0000427722 | FP: TCCGTTCGCCTTATTACA  RP: GTTAGGACGCACTTGAAG | 59.8  59.5 | 155 |
| Pathogenesis-related thaumatin superfamily protein | MDP0000246775 | FP: TCAACTTGCCTATGTCTGT  RP:CAACTGATGACACTCCCA | 60.2  60.4 | 131 |
| Homolog of carrot EP3-3 chitinase | MDP0000702868 | FP: TTCCTATACTCAGTTCGGTAG  RP:GGTCTCATCACAGTAGTCTT | 59.9  60.2 | 138 |
| Chitinase A | MDP0000888042 | FP: GCTCTCAAGTCACAAGTTC  RP:GATGCTGTCCTTAATGGATG | 60.0  60.1 | 113 |
| Glutathione S-transferase | MDP0000279981 | FP: CATTGCTGCTCTTCTTAGG  RP:CTCCTCTTCGGTCTCTTC | 59.8  59.6 | 200 |
| Glycosyl hydrolase superfamily protein | MDP0000570395 | FP: CCTATGCCTTGTTCACTTC  RP:TTGCTTGATTTCCACCTTC | 59.7  59.9 | 174 |
| NAD dependent epimerase/ dehydratase family protein | MDP0000193729 | FP: CCAACCATTGTTCCTTCAG  RP: CTTCACCAACTGTCAAGAC | 59.9  60.3 | 159 |

**Supplementary Table S2. Summary of RNA-seq and mapping results**

Table S2-1.Summary of mapping result (mapping to reference genome)

| Sample ID | NO. of raw reads | NO. of mapped reads | NO. of perfect match reads | NO. of <=2bp mismatch reads | NO. of unique match reads | NO. of multi-position match reads |
| --- | --- | --- | --- | --- | --- | --- |
| 0 HPI | 11,960,715(100%) | 10,122,347(84.63%) | 7,388,873(61.78%) | 2,733,474(22.85%) | 6,562,337(54.87%) | 3,560,010(29.76%) |
| 12 HPI | 12,613,214(100%) | 10,699,158(84.82%) | 7,792,386(61.78%) | 2,906,772(23.05%) | 6,906,178(54.75%) | 3,792,980(30.07%) |
| 18 HPI | 12,002,360(100%) | 10,196,807(84.96%) | 7,443,626(62.02%) | 2,753,181(22.94%) | 6,541,429(54.50%) | 3,655,378(30.46%) |
| 36 HPI | 12,428,635(100%) | 10,521,962(84.66%) | 7,658,309(61.62%) | 2,863,653(23.04%) | 6,773,429(54.50%) | 3,748,533(30.16%) |
| 72 HPI | 12,360,687(100%) | 10,030,787(81.15%) | 7,307,049(59.12%) | 2,723,738(22.04%) | 6,395,886(51.74%) | 3,634,901(29.41%) |

Table S2-2.Summary of mapping result (mapping to reference genes)

| Sample ID | NO. of raw reads | NO. of mapped reads | NO. of perfect match reads | NO. of <=2bp mismatch reads | NO. of unique match reads | NO. of multi-position match reads |
| --- | --- | --- | --- | --- | --- | --- |
| 0 HPI | 11,960,715(100%) | 7,894,402(66.00%) | 5,936,687(49.63%) | 1,957,715(16.37%) | 5,449,730(45.56%) | 2,444,672(20.44%) |
| 12 HPI | 12,613,214(100%) | 8,358,147(66.27%) | 6,255,434(49.59%) | 2,102,713(16.67%) | 5,449,730(45.56%) | 2,649,654(21.01%) |
| 18 HPI | 12,002,360(100%) | 7,932,009(66.09%) | 5,924,343(49.36%) | 2,007,666(16.73%) | 5,410,132(45.08%) | 2,649,654(21.01%) |
| 36 HPI | 12,428,635(100%) | 8,199,868(65.98%) | 6,100,485(49.08%) | 2,099,383(16.89%) | 5,608,951(45.13%) | 2,590,917(20.85%) |
| 72 HPI | 12,360,687(100%) | 7,827,010(63.32%) | 5,805,659(46.97%) | 2,021,351(16.35%) | 5,378,852(43.52%) | 2,448,158(19.81%) |
